# Early Adolescents’ Social Achievement Goals and Perceived Relational Support: Their Additive and Interactive Effects on Social Behavior

**DOI:** 10.3389/fpsyg.2021.767599

**Published:** 2021-12-06

**Authors:** Huiyoung Shin

**Affiliations:** Department of Psychology, Jeonbuk National University, Jeonju, South Korea

**Keywords:** social achievement goals, relational support, aggression, anxious solitude, prosocial behavior, early adolescence

## Abstract

The current study examined the additive and interactive effects of early adolescents’ social achievement goals and perceived relational support from teachers and peers on their social behavior. Adolescents’ social achievement goals (i.e., social development, social demonstration-approach, and social demonstration-avoidance), perceived relational support from teachers and peers, and social behavior (i.e., overt and relational aggression, prosocial behavior, and anxious solitary behavior) were assessed in a sample of fifth and sixth graders (*M*_age_ = 12.5; *N* = 677) nested within 26 classrooms. Multilevel modeling results indicated that social goals and relational support from teachers and peers made additive contributions to adolescents’ social behavior. Results also indicated the evidence of interactive effects, such that relational support from teachers was negatively associated with overt and relational aggression primarily among adolescents who had high social demonstration-approach goals. Findings underscore the need to consider adolescents’ social goals in conjunction with their perceived relational support for educators and practitioners.

## Introduction

Research on social goals has received much attention in recent decades of research. Scholars have drawn attention to more than a dozen different types of social goals that youth pursue with peers (Jarvinen and Nicholls, 1996; Rose and Asher, 1999; Wentzel, 2001) and have highlighted the importance of youth’s social goals in their peer relations and social adjustment (Ojanen et al., 2012; Shin and Ryan, 2012; Kiefer and Shim, 2016). Social goals are cognitive representations of things that individuals want to accomplish in the social domain, and provide direction and energy for their behavior in the social relationship (Ryan and Shim, 2008). Whether youth are development or demonstration (or agency or communion) oriented in their social relationships is an important distinction that has shown different linkages with their social behavior (Ojanen et al., 2005; Ryan and Shim, 2006). For instance, adolescents’ demonstration-approach (or dominance) goals are positively associated with aggression, whereas development (or closeness) goals are positively associated with prosocial behavior (Ryan and Shim, 2008; Ojanen and Fineley-Van Nostrand, 2014).

Although the linkages between social goals and social behavior have been extensively examined, scant attention has been paid to the role of social relatedness as a possible moderator. Thus, it remains mostly unclear if the role of social goals on adolescents’ social behavior is contextualized by relational support from teachers and peers, which are major social relatedness features for early adolescents. Because interactions with teachers and peers are a salient feature of school, their relational support is likely to affect the associations between adolescents’ social goals and their social behavior. Indeed, youth’s relationships with teachers and peers have been found to make additive or contingent contributions to youth’s social adjustment outcomes such as aggression, behavioral misconduct, and prosocial behavior (e.g., Wentzel et al., 2016; Shin et al., 2019).

The aim of the current study is to examine if youth’s social achievement goals and perceived relational support from teachers and peers have the additive and interactive effects on their social behavior. Two main goals are to examine the extent to which social goals and perceived relational support make additive contributions to youth’s subsequent social behavior and whether the linkages between social goals and social behavior are moderated by perceived relational support. These goals were addressed using a prospective longitudinal design in which youth evaluate their social goals, social behavior, and perceptions of relational support from both teachers and peers from the adolescent’s perspective. With considering the full social context that youth experience in class, the current study will substantiate a more multifaceted understanding on the nature and implications of perceived relational support. Findings on the associations of youth’s social goals and relational experiences youth have with teachers and peers, and how they pertain to youth’s social behavior will provide important implications for educators and practitioners.

### Social Achievement Goals and Social Behavior

Research on social goals has provided insights into adolescents’ different behavior and adjustment at school (Caravita and Cillessen, 2012; Kiefer and Shim, 2016). Among different approaches to the conceptualization of social goals, one promising approach has been to examine the social goal orientations. Research applying the social goal orientations approach has examined three different goal orientations youth have toward achieving social competence: social development, social demonstration-approach, and social demonstration-avoidance. These different social goal orientations capture meaningful distinctions in how individuals orient themselves toward forming and maintaining social relationships (Ryan and Shim, 2006).

Social development goals focus on developing social competence. With social development goals, youth use intrapersonal standards to evaluate their social competence. The focus is on whether they are having the growth of social relationships, improving interpersonal skills, or developing their social life in general (Ryan et al., 2012). Since these youth has positive views about their social competence and enhanced efficacy in social interactions, many social challenges provide opportunities for developing social skills and close friendships. They believe that they can improve and grow in positive ways in their relationships and thus mistakes are not threatening which decreases anxiety around peers. With their goals of developing positive friendships, adolescents attend to cues regarding the compatibility with other peers and be thoughtful about what is best for their relationships (Shin and Ryan, 2012). Thus, social development goals are positively associated with intimacy and mutual support as well as prosocial behavior and negatively associated with solitary or aggressive behavior with peers (Ryan et al., 2012).

In contrast, social demonstration goals focus on demonstrating social competence. Social demonstration-approach goals focus on gaining positive judgments from others (e.g., garnering social prestige and positive evaluation), whereas social demonstration-avoidance goals focus on avoiding negative judgments from others (e.g., being seen as socially ineffective or awkward). With both social demonstration goals, youth use interpersonal standards to judge their social competence which concern social comparisons with other peers. With the inherent “approach” nature of social demonstration goals, youth with social demonstration-approach goals are both adaptive and maladaptive in beliefs and behaviors. They generally feel socially efficacious and strive to achieve social status and recognition among peers. However, their behavior strategies focus on social appearance and impression making rather than relationship building (Rodkin et al., 2000). And with the goals to achieve desired social status, their behavioral tactics tend to include social manipulation and overt aggression (Rodkin et al., 2013). Thus, social demonstration-approach goals are positively associated social status among peers such as being popular but negatively associated with developing positive qualities in friendships (Shin, 2017).

With the focus on avoiding negative social judgments, youth with social demonstration-avoidance goals experience maladaptive beliefs and behaviors. These youth generally have negative views of their social competence, fear of failure, and diminished efficacy to achieve desired social outcomes (Ryan et al., 2012). Thus, they usually “withdraw” from engaging in social interactions as it is safer and satisfies their goals of avoiding possible negative social outcomes. Inherent in social demonstration-avoidance goals are the belief that other peers’ judgments determine youth’s social success or failure. For youth with these goals, mistakes or misunderstandings in social interactions would incur negative evaluations and are therefore threatening. Due to a focus on negative social outcomes and being overly self-conscious and afraid of failure, their peer relationships suffer with increased anxious behavior and avoidant strategies (Kuroda and Sakurai, 2011; Shin and Ryan, 2012). Thus, social demonstration-avoidance goals undermine the development of close friendships as well as social status among peers, and are associated with loneliness and isolation (Mouratidis and Sideridis, 2009; Liem, 2016).

### Social Achievement Goals and Social Behavior Among Asian Adolescents

Research on Asian adolescents’ social achievement goals is lacking compared to youth in the Western populations. Although the theoretical conceptualizations of social achievement goal orientations and the associations between social goals and social behavior are presumed to be universal in nature, the salience of different goals and whether certain goals are more adaptive or maladaptive for Asian youth is arguably not certain (Makara, 2019). For youth who are in the cultural orientation of individualism, one’s well-being is linked to the attainment of one’s personal goals, whereas youth who are in the cultural orientation of collectivism focus on group membership and interdependence (Oyserman et al., 2002). Asian countries such as China, South Korea, and Japan tend to have collectivist cultural orientations, in contrast to the United States or European countries which tend to have individualistic cultural orientations (Hofstede, 2001).

Given these differences, it is likely that collectivist or individualistic cultural orientations may have implications for what type of social goals youth pursue and the consequences of endorsing particular social goals. For example, it could be assumed that social development goals may be more dominant in collectivist cultures due to the focus on others more than the self (Makara, 2019). And, social demonstration-avoidance goals could be more maladaptive for Asian youth considering their emphasis on harmonious relationships and interdependence (Markus and Kitayama, 1991). Indeed, in a study with Japanese early adolescents, Kuroda and Sakurai (2011) reported that social development goals reduced the effects of interpersonal stress and protected youth again depression, whereas social demonstration-avoidance goals exacerbated youth’s interpersonal stress and depression. However, a lack of cross-cultural research on social achievement goals make it difficult to make comparisons between studies to explore whether similar patterns emerge or not. Using an expanded set of social behavior that characterizes adolescents’ social-behavioral orientations as moving toward, away from, or against the social world (i.e., prosocial behavior, anxious solitary behavior, and aggression; Caspi et al., 1988) based on South Korean early adolescents, the current study will provide additional evidence about the nature and the associations between social goals and social behavior.

### Social Achievement Goals, Relational Support, and Social Behavior

Person × Environment (P × E) models emphasize that youth’s personal characteristics (e.g., social goal orientations) in conjunction with environmental factors (e.g., relational support) jointly affect their developmental trajectories (Ladd, 2003). Although several variants of P × E models have been suggested to elucidate the distinct and conjoint contributions of youth’s personal characteristics and relational factors to their adjustment (Ettekal and Ladd, 2017; Ladd et al., 2019), most of the empirical evidence amassed conforms to additive models that consider main effects of social goals on social behavior. Thus, evidence is not sufficient if the role of social goals on social behavior is contextualized by relational supports or stressors for early adolescents. In view of these limitations, the current study considers both additive and interactive models. Additive models imply that, separate from the contributions of youth’s social goals, the relational experiences youth have with teachers or peers are positively or negatively associated with their social behavior. Alternatively, interactive models imply that contributions of youth’s social goals to social behavior are contingent on the levels of relational experiences that youth perceive and are therefore moderate the effects of social goals on social behavior.

Prior theory and research support the contention that youth vary in the closeness and relatedness of their relationships with teachers and peers (Furrer and Skinner, 2003; Hughes et al., 2014), and personal relational experiences operate as relational supports or stressors in the social context (Ladd et al., 1997). According to self-determination theory (SDT), relatedness is one of the fundamental psychological needs that must be fulfilled (Deci and Ryan, 2000). When youth’s psychological need for social relatedness is met, it sets in motion self-system processes that promote positive behavior in the social setting (Connell and Wellborn, 1991). Ryan and Deci (2000) emphasized the social conditions that foster or impede internalization or integration of behavior in SDT. That is, youth engage in behaviors for others when those behaviors are prompted, valued, or modeled by significant others to whom they feel related and attached. Thus, when youth feel close to and supported by their teachers and peers, they would be motivated to comply with the teacher’s expectations and act in ways that are valued by their peers.

Indeed, there is compelling empirical evidence that relational support from teachers and peers contributes to emerging patterns of social behavior. Relational support from teachers, characterized by warmth, closeness, and open communication, empowers youth to engage in, rather than withdraw from, social activities in the classroom (Gest et al., 2005). When youth perceived their teachers as supportive and involved and were provided with feelings of caring and encouragement, perceived relational support were positively associated with youth’s prosocial behavior and were negatively associated with aggression (Luckner and Pianta, 2011; Shin et al., 2019). In a similar manner, perceived relatedness from peers function as support systems that facilitate youth’s social adaptation (Wentzel et al., 2018). Psychological processes such as validation, self-disclosure, and emotional and instrumental help are prominent among friends, and these supportive processes are directly linked with youth’s social adaptation (Ladd et al., 1997). In contrast, perceived conflict or hostile interactions works as an impediment to social adaptation because it leads to social alienation and exclusion and restricts youth’s access to social activities (Shin, 2019). When youth experienced rejection or victimization by peers, due to its exploitive nature, it amplified youth’s loneliness and anxieties as well as irresponsible behavior and dampened social competence and prosocial behavior (Wentzel, 2003; Kingery and Erdley, 2007). Therefore, consistent with theory and empirical findings, it is anticipated that youth’s perceived relational support from teachers and peers would make additive contributions to their aggression, prosocial behavior, and anxious solitary behavior.

In addition to the additive effects, relational support from teachers and peers are expected to have interactive effects. That is, the contribution of social goals to social behavior could be moderated by the levels of perceived relatedness. Support for this premise includes previous evidence indicating that the levels of relational support exacerbates or compensates for dysfunctions that are linked with risk factors such as aggression and isolation. For example, positive relationships with teachers attenuated aggressive youth’s subsequent aggression while conflicted relationships with teachers were more predictive of aggressive youth’s more chronic aggression (Hughes et al., 1999). Further, conflicts with peers or teachers strengthened the linkages between aggression and emerging patterns of maladjustment such as increasing misconduct and declining cooperation (Ladd and Burgess, 2001).

Among youth with social demonstration goals, perceived relatedness may temper the linkages between social demonstration goals and aggression or anxious solitary behavior. Additionally, adolescents’ perceived relatedness may have stronger buffering effects for youth who have higher rather than lower levels of social demonstration goals. Such moderated linkages could be anticipated if the processes afforded by perceived relatedness serve to counteract the risks posed by social demonstration goals. For example, if youth with high social demonstration goals perceive that a teacher likes them and cares about them as an individual, they would have fostered feelings of social confidence and self-worth, and be more influenced by teachers’ values and expectations (SDT; Ryan and Deci, 2000). Accordingly, their attention to the appearance of the self or concern with others’ judgments, strivings to achieve social status or recognition using aggressive actions as well as withdrawal from engaging in social interactions to avoid negative evaluations are likely to diminish (Connell and Wellborn, 1991). In contrast, if youth with high social demonstration goals perceive conflict or rejection from teachers or peers, it would magnify youth’s negative emotions such as fearfulness and anxiety, and cause youth to further withdraw from social activities (Ettekal and Ladd, 2020). These youth may be prone to further develop maladaptive social cognitions and become enmeshed in cycles of aggression or isolation. In the current study, this possibility was addressed by examining the degree to which perceived relational support from teachers and peers moderated the magnitude of the associations between adolescents’ social goals and their subsequent social behavior.

### Gender and Grade

Previous research indicates that boys and girls may differ in their social goal orientations, nature of relationships, and social behavior. Girls tend to be more oriented toward deepening the quality of friendships whereas boys tend to be more oriented toward displaying social competence (Rose and Rudolph, 2006). Thus, boys are more likely to emphasize and communicate assertiveness and dominance (Shin, 2017) and tend to use more overt aggression compared with girls (Dijkstra et al., 2009). In contrast, prosocial behavior is often more prevalent among girls compared with boys (Van der Graaff et al., 2017). Furthermore, boys are less likely than girls to have close and positive relationships with their teachers and peers (Jerome et al., 2009). In general, girls’ friendships tend to be characterized by higher levels of intimacy, support, and self-disclosure than boys (Parker and Asher, 1993). In light of this evidence, potential gender differences in the levels of social behavior as well as the associations between social goals, relational support, and social behavior can be expected.

Also, important developmental changes during early adolescence may affect the nature and the associations between social goals and social behavior. Peer climate changes in ways that approve deviance and aggression, and depress compliant and prosocial behavior (Cillessen and van den Berg, 2012). Thus, at this stage, aggressive behavior is often evaluated with more positive light (Galvan et al., 2011). Also, as children enter adolescence, they become more adept at social skills and strategies, and thus may use less discernable aggressive behavior. They use more covert forms of aggression such as relational aggression as an appropriate way to maneuver their peer relationships (Godleski and Ostrov, 2010). With increased levels of self-consciousness and sensitivity to feedback from peers (Steinberg, 2014), the salience of certain social goals and the associations between social goals and aggression, especially relational aggression, may be amplified during early adolescence. Thus, in the current study, potential gender differences in the mean level of research variables as well as the moderating role in any of the individual level associations will be explored with the sample of early adolescents (i.e., fifth and sixth graders in elementary school).

### Overview of the Current Study

The current study used a prospective longitudinal design to examine if early adolescents’ social goals and perceived relational support from teachers and peers have the additive and interactive effects on their social behavior. One objective was to determine whether early adolescents’ social goals and perceived relational support from teachers and peers were additively associated with their subsequent social behavior. A related objective was to ascertain whether the associations between social goals and social behavior were contingently altered by perceived relational support from teachers and peers. In general, it was expected that the contribution of social goals and perceived relational support to social behavior would be additive. It was hypothesized that social development goals and perceived relational support would be positively associated with prosocial behavior, whereas social demonstration goals and perceived negative relational support would be positively associated with overt and relational aggression as well as anxious solitary behavior. In addition, it was expected that perceived relational support would mitigate the associations between social demonstration goals and aggression or anxious-solitary behavior.

## Materials and Methods

### Participants and Procedures

Participants were fifth- and sixth-grade students from public elementary schools in South Korea. In South Korea, the elementary schools contain first- to sixth-grades, and students stay in a same classroom with a teacher and the peers for the entire school day. For the current study, students participated in the research when they began (Wave 1: August) and at the end (Wave 2: December) of their second semester. The students’ parents received a letter explaining what was involved in participating in the research, and if they did not want their children to participate, they could opt out by contacting the school; otherwise, students took part in the study. Students were informed that their participation was voluntary and that their responses would be kept confidential, and they signed an assent form indicating that they understood the conditions and wanted to participate prior to starting the survey. In order to make model comparisons, students who participated in both waves were included, which results in a final sample of 677 students nested within 26 classrooms (339 fifth graders, 48% male, *M*_age_ = 12.46).

### Measures

Consistent with our conceptualization that social achievement goals precede social behavior, we used a prospective longitudinal design to investigate whether social goals foreshadow subsequent social behavior (5-month time span). Self-reported measures of social achievement goals, relational support from teachers and peers were assessed in Wave 1. Self-reported measures of overt aggression, relational aggression, prosocial behavior, and anxious solitary behavior were assessed in Wave 2. All self-reported measures described below used a five-point scale that ranged from 1 to 5. Since we used self-reported measures for all research variables, we checked the common method biases using Harman’s single factor score, in which all items are loaded into one common factor, to assuage concerns about the possibility of common method effects underlying observed results. If the total variance extracted by one factor exceeds 50%, it suggests that common method bias is present and affect the results (Podsakoff et al., 2012). In our data, the total variance extracted by one factor was 17.74% and it is less than the recommended threshold of 50%. Thus, there was no problem with common method bias in our data.

#### Social Achievement Goals

Three social achievement goals were measured with Ryan and Shim (2008)’s scale for early adolescents. Social development goal items focus on developing youth’s social competence (e.g., “One of my goals is to be a better friend to others”). Social demonstration-approach goal items focus on demonstrating youth’s social desirability and gaining positive evaluations from others (e.g., “I try to do things that make me look good to others”). Social demonstration-avoidance goal items focus on avoiding negative judgments from others (e.g., “It is important to me that I don’t embarrass myself around my friends”). The measure was consisted of eighteen items (six items for each social goal orientation) and students were instructed to report on a scale that ranged from 1 (*not at all true of me*), 3 (*somewhat true of me*), to 5 (*very true of me*) for all items. The average score of the items was computed, with higher scores indicative of higher goals. The scale was reliable in the current sample (α = 0.88, 0.88, and 0.80 for social development, social demonstration-approach, and social demonstration-avoidance goals, respectively).

#### Teacher Support

Students’ perceived social support from their teacher was assessed using the teacher social support subscale of the Classroom Life Measure (Johnson et al., 1983). The measure was consisted of four items and sample items are “My teacher tries to help me when I am sad or upset” and “I can count on my teacher for help when I need it.” Students were instructed to report on a scale that ranged from 1 (*never*), 3 (*sometimes*), to 5 (*always*) for all items. The average score of the items was computed, with higher scores indicative of higher teacher support. This scale was reliable in the current sample (Cronbach’s α = 0.86).

#### Peer Support

Students’ perceived peer support was measured using the negative peer interaction subscale of the Inventory of School Climate-Student (Brand et al., 2003). The measure was consisted of five items and sample items are “Students are often teased or picked on,” “Most students are friendly to each other.” Students were instructed to report on a scale that ranged from 1 (*never*), 3 (*sometimes*), to 5 (*always*) for all items. Negative items were reverse-coded. The average score of the items was computed, with higher scores indicative of higher peer support. This scale was reliable in the current sample (Cronbach’s α = 0.78).

#### Overt Aggression

Students’ overt aggression referred to physical or verbal acts of aggressive behavior. To assess the overt aggression, the student-report version of the Aggression subscale of the Interpersonal Competence Scale (Cairns et al., 1995) was used. It was consisted of three items: “I fight with others,” “I argue with others,” and “I get in trouble.” Students were instructed to report on a scale that ranged from 1 (*never*), 3 (*sometimes*), to 5 (*always*) for all items. The average score of the items was computed, with higher scores indicative of higher overt aggression. This scale was reliable in the current sample (Cronbach’s α = 0.80).

#### Relational Aggression

Students’ relational aggression referred to relationship manipulation including acts of gossiping and social exclusion, and was measured using the Children’s Social Behavior Scale (Crick, 1996). It was consisted of four items and sample items are “I ignore some friends or stop talking to them,” and “I try to keep certain friends from being in my group.” Students were instructed to report on a scale that ranged from 1 (*never*), 3 (*sometimes*), to 5 (*always*) for all items. The average score of the items was computed, with higher scores indicative of higher relational aggression. This scale was reliable in the current sample (Cronbach’s α = 0.83).

#### Prosocial Behavior

Students’ prosocial behavior referred to cooperative and help-providing behavior. To assess the prosocial behavior, the measure from Cassidy and Asher (1992) and Crick (1996) was used. It was consisted of five items and sample items are “I help others,” “I am considerate of others’ feelings,” and “I am kind to others.” Students were instructed to report on a scale that ranged from 1 (*never*), 3 (*sometimes*), to 5 (*always*) for all items. The average score of the items was computed, with higher scores indicative of higher prosocial behavior. This scale was reliable in the current sample (Cronbach’s α = 0.82).

#### Anxious Solitary Behavior

Students’ anxious solitary behavior was assessed using Gazelle’s measure (Gazelle and Ladd, 2003; Gazelle and Rudolph, 2004). The measure was consisted of eight items and sample items are “I play alone more than most peers,” “I am self-conscious or easily embarrassed,” “I am shy or timid,” and “I am anxious around peers.” Students were instructed to report on a scale that ranged from 1 (*not true*), 3 (*sometimes true*), to 5 (*always true*) for all items. The average score of the items was computed, with higher scores indicative of higher anxious solitary behavior. This scale was reliable in the current sample (Cronbach’s α = 0.82).

### Analytic Strategy

Multilevel modeling was used due to the nested nature of the observed data. Two-level models (i.e., students nested within 26 classrooms) were estimated in R 4.0.3 with the nlme package (v3.1-152; Pinheiro et al., 2021). Separate parallel models were built to examine the contributions of social achievement goals and relational support from teachers and peers on subsequent four different social behavior (see the equation in the Supplementary Appendix). Gender and grade were included as level 1 covariates in each model. When gender and grade were included in the multilevel models, responses were dummy coded such that zeros reflected males and fifth graders, while ones reflected females and sixth graders, respectively. To create classroom level relational support, students’ individual perceptions of teacher support and peer support were aggregated to create a level 2 mean score for each classroom (variability between classrooms), while students’ individual reports (representing their personal perceived relational support) were retained at level 1 (variability within classrooms). All student-level variables (Level 1) were classroom group-mean centered and classroom-level variables (Level 2) were grand-mean centered.

Multilevel models were built beginning with the null models before adding student-level variables (i.e., social achievement goals, individual perceived relational support) and level 1 interactions, and then proceeding to level 2 main effects and exploring cross-level interactions (Snijders and Bosker, 2012). The first model examined student-level associations between the independent and dependent variables, beginning with students’ gender and grade, followed by social achievement goals, individual-level relational support, and interactions between independent variables (e.g., social achievement goals × teacher support, gender × teacher support). Then, at the classroom-level, following model examined between classroom differences in relational support by adding both the level 1 and level 2 relational support from teachers and peers. Next, cross-level interactions between classroom-level and individual-level variables (e.g., teacher support mean × social achievement goals, teacher support mean × gender) were examined. Only significant interaction terms from the full model were retained in the final model and simple slope tests were conducted for significant interaction (Preacher et al., 2006). The multilevel equation representing the full model that was estimated for students’ social behavior is presented in the Supplementary Appendix.

## Results

### Descriptive Statistics

Bivariate correlations for social achievement goals, relational support from teachers and peers, and social behavior are presented in Table 1. For the most part, an expected pattern was found among the variables. Social development goals were negatively associated with overt and relational aggression but positively associated with prosocial behavior. Social demonstration-approach goals were positively associated with overt and relational aggression, whereas social demonstration-avoidance goals were positively associated with anxious solitary behavior. Both teacher support and peer support were negatively associated with overt and relational aggression as well as anxious solitary behavior but positively associated with prosocial behavior.

**TABLE 1:** Correlations among social goals, relational support from teachers and peers at Wave 1, and social behavior at Wave 2.

	**1**	**2**	**3**	**4**	**5**	**6**	**7**	**8**
**Wave 1**								
1. Social development								
2. Social demonstration-approach	0.38***							
3. Social demonstration-avoidance	0.47***	0.42**						
4. Teacher support	0.29***	0.14***	0.17***					
5. Peer support	0.05	0.05	0.02	0.24***				
**Wave 2**								
6. Overt aggression	−0.08*	0.09*	–0.01	−0.12**	−0.11**			
7. Relational aggression	−0.10*	0.09*	–0.03	−0.18**	−0.21***	0.58***		
8. Prosocial behavior	0.34***	0.13**	0.19**	0.28***	0.09*	−0.14**	−0.18**	
9. Anxious solitary behavior	0.01	0.08*	0.18**	−0.11**	−0.18**	0.45**	0.45**	0.01

*p < 0.05, **p < 0.01, ***p < 0.001.

The means and standard deviations as well as gender and grade differences for all variables are presented in Table 2. Girls reported higher social development goals (*t* = −5.57, *p* < 0.001), social demonstration-approach goals (*t* = −2.52, *p* < 0.05), prosocial behavior (*t* = −3.37, *p* < 0.001), and anxious solitary behavior (*t* = −3.01, *p* < 0.01) than boys, whereas boys reported higher overt aggression (*t* = 4.09, *p* < 0.001) than girls. Sixth graders reported higher relational aggression (*t* = −2.28, *p* < 0.05) than fifth graders.

**TABLE 2:** Means and standard deviations of social goals, relational support from teachers and peers, and social behavior.

		**All**	**Boys**	**Girls**		**5th**	**6th**	
	***N***	***M* (*SD*)**	***M* (*SD*)**	***M* (*SD*)**	***t***	***M* (*SD*)**	***M* (*SD*)**	***t***
**Wave 1**								
Social development	618	4.12(0.66)	3.97(0.72)	4.26(0.57)	−5.57***	4.13(0.65)	4.11(0.68)	0.25
Social demonstration-approach	627	2.94(0.90)	2.84(0.88)	3.02(0.92)	−2.52*	2.93(0.95)	2.94(0.85)	–0.10
Social demonstration-avoidance	626	3.60(0.77)	3.56(0.76)	3.64(0.78)	–1.28	3.65(0.75)	3.55(0.79)	1.52
Teacher support	622	3.20(0.90)	3.15(0.89)	3.24(0.90)	–1.31	3.22(0.88)	3.17(0.90)	0.61
Peer support	623	2.11(0.70)	2.16(0.71)	2.07(0.69)	–1.67	2.09(0.70)	2.14(0.70)	0.91
**Wave 2**								
Overt aggression	677	2.51(0.86)	2.65(0.88)	2.38(0.82)	4.09***	2.49(0.84)	2.54(0.88)	–0.81
Relational aggression	677	2.29(0.87)	2.29(0.87)	2.29(0.88)	–0.13	2.21(0.87)	2.36(0.87)	−2.28*
Prosocial behavior	677	3.42(0.76)	3.32(0.76)	3.51(0.74)	−3.37***	3.41(0.80)	3.43(0.72)	–0.22
Anxious solitary behavior	677	2.62(0.83)	2.52(0.83)	2.71(0.83)	−3.01**	2.57(0.83)	2.68(0.82)	–1.78

*Ranges for all variables were 1–5.*

*p < 0.05, **p < 0.01, ***p < 0.001.

### Multilevel Modeling Results

Unconditional multilevel models were first investigated to examine how much variability existed within and between classroom levels for research variables. Excluding the non-significant intraclass correlation for anxious solitary behavior (less than 1%), overt aggression exhibited the lowest variability between classrooms (3%) followed by prosocial behavior (4%) and relational aggression (9%). The sizeable proportion of variance occurred at the classroom-level for teacher support (13%) and peer support (7%). Social achievement goals exhibited the lowest variability between classrooms (social development = 2%, social demonstration-approach = 1%, social demonstration-avoidance = 5%, respectively). Based on these analyses, the within classroom (Level 1) associations between students’ gender and grade, social achievement goals, individual-level relational support and their levels of social behavior were examined. Preliminary models explored all possible interaction effects among individual level variables, and only significant interaction terms (i.e., social demonstration-approach goals × teacher support, teacher support × gender) were retained in the models. Then, by additionally incorporating relational support at the classroom-level means (Level 2), following models examined the main effects associated with between classroom differences in relational support after all individual level variables have been accounted for. When relational support from teachers and peers from both levels were included, teacher support at level 2 was not significant for all social behavior, and peer support at level 2 was significantly associated with only prosocial behavior. Next, the last model examined all possible cross-level interactions between classroom-level and individual-level variables in the full model. However, all of these cross-level interactions were not significant and thus only the main effects of the classroom level relational support from teachers and peers were retained in the final model. The final results for each social behavior are presented in Table 3 and are described below.

**TABLE 3:** Multilevel models predicting social behavior at Wave 2 from individual- and class-level variables at Wave 1.

	**Overt aggression β (*SE*)**	**Relational aggression β (*SE*)**	**Prosocial behavior β (*SE*)**	**Anxious solitary behavior β (*SE*)**
**Fixed effects**				
Intercept	0.41(0.20)*	−0.19(0.24)	−0.24(0.19)	−0.49(0.19)*
**Level 1**				
Gender^a^	−0.34(0.08)***	0.03 (0.07)	0.15 (0.08)	0.21(0.08)*
Grade^b^	0.07 (0.10)	0.15 (0.14)	−0.01(0.09)	0.11 (0.09)
Social development	−0.04(0.05)	−0.10(0.04)*	0.25(0.04)***	−0.12(0.05)*
Social demonstration-approach	0.15(0.04)**	0.16(0.04)***	−0.03(0.04)	0.04 (0.04)
Social demonstration-avoidance	−0.03(0.05)	−0.01(0.04)	0.06 (0.04)	0.22(0.05)***
Teacher support	−0.41(0.15)***	−0.10(0.04)*	0.19(0.04)***	−0.10(0.05)*
Peer support	−0.13(0.04)**	−0.19(0.04)***	0.02 (0.04)	−0.19(0.04)***
Demonstration-approach × teacher support	−0.10(0.03)*	−0.11(0.03)**		
Teacher support × gender	0.21(0.09)*			
**Level 2**				
Teacher support mean	−0.06(0.16)	−0.26(0.21)	0.10 (0.15)	−0.05(0.15)
Peer support mean	−0.01(0.19)	−0.15(0.26)	0.35(0.19)*	−0.10(0.19)
**Random effects**				
Student-level variance	0.68	0.65	0.47	0.61
Classroom-level variance	0.03	0.06	0.02	0.01
**Model fit**				
−2Log likelihood	−854.21	−843.44	−733.36	−817.22
AIC	1736.42	1712.89	1490.72	1658.44

*^*a*^Gender is coded 0 = *male* and 1 = *female*.*

*^*b*^Grade is coded 0 = *5th grader* and 1 = *6th grader*.*

*p < 0.05, **p < 0.01, ***p < 0.001.

#### Overt Aggression

**FIGURE 1:**
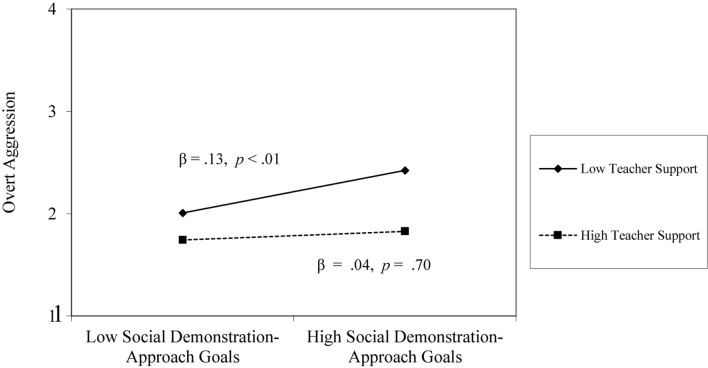
Social demonstration-approach goals × teacher support interaction on overt aggression.

**FIGURE 2:**
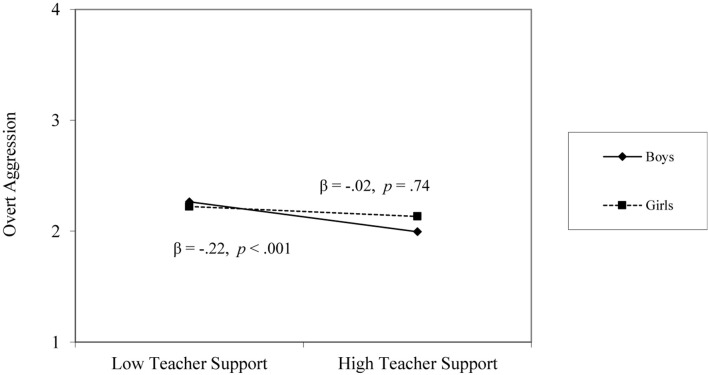
Teacher support × gender interaction on overt aggression.

At the individual level, gender, social demonstration-approach goals, and relational support from teacher and peers were significantly associated with overt aggression. Girls reported lower levels of overt aggression than boys (β = −0.34, *p* < 0.001), and social demonstration-approach goals were positively associated with overt aggression (β = 0.15, *p* < 0.01), such that students with high social demonstration-approach goals reported higher levels of overt aggression. Perceived teacher support and peer support demonstrated the strong negative associations with overt aggression (β = −0.41, *p* < 0.001; β = −0.13, *p* < 0.01). There was a significant interaction between social demonstration-approach goals and perceived teacher support, such that the positive association between social demonstration-approach goals and overt aggression was only found when youth perceived low teacher support (β = 0.13, *p* < 0.01); there was no significant relation when youth perceived high teacher support (β = 0.04, *p* = 0.70; see Figure 1). Further, there was a significant interaction between perceived teacher support and gender: The influence of perceived teacher support on overt aggression was only found for boys (β = −0.22, *p* < 0.001); the influence of perceived teacher support on overt aggression was not significant for girls (β = −0.02, *p* = 0.74; see Figure 2). To calculate changes in variance that these variables accounted for, the unexplained variance in the final model from the null model was subtracted and divided by the total variance. Together, these individual-level variables reduced the unexplained variance by 6%. At the classroom-level, classroom-level means for teacher support and peer support were unrelated to students’ overt aggression (β = −0.06, *p* = 0.88; β = 0.01, *p* = 0.78).

#### Relational Aggression

**FIGURE 3:**
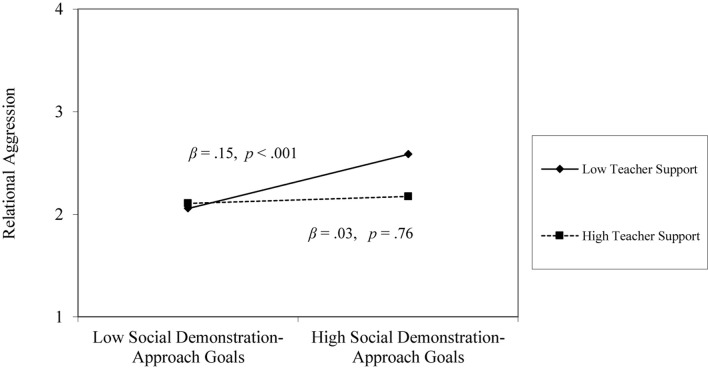
Social demonstration-approach goals × teacher support interaction on relational aggression.

At the individual level, social development and social demonstration-approach goals, and relational support from teachers and peers were significantly associated with relational aggression. Social development goals were negatively associated with relational aggression (β = −0.10, *p* < 0.05), and social demonstration-approach goals were positively associated with relational aggression (β = 0.16, *p* < 0.001). Perceived teacher support and peer support demonstrated the negative associations with relational aggression (β = −0.10, *p* < 0.05; β = −0.19, *p* < 0.001). There was a significant interaction between social demonstration-approach goals and perceived teacher support. Similar to the patterns that were found with overt aggression, the positive association between social demonstration-approach goals and relational aggression was only found when youth perceived low teacher support (β = 0.15, *p* < 0.001); there was no significant relation when youth perceived high teacher support (β = 0.03, *p* = 0.76; see Figure 3). Students’ individual-level variables reduced the unexplained variance by 7% from the null model. At the classroom-level, similar to the patterns that were found with overt aggression, classroom-level means for teacher support and peer support were unrelated to students’ relational aggression (β = −0.26, *p* = 0.15; β = −0.15, *p* = 0.56).

#### Prosocial Behavior

At the individual level, social development and relational support from teachers were significantly associated with prosocial behavior. Social development goals were positively associated with prosocial behavior (β = 0.25, *p* < 0.001), and perceived teacher support was positively associated with prosocial behavior (β = 0.19, *p* < 0.001). Results for the classroom-level indicated that students’ collective perceptions of peer support were positively associated with students’ prosocial behavior (β = 0.35, *p* < 0.05). Therefore, the classroom level peer support provides a unique contribution that explains variability in students’ prosocial behavior in addition to students’ individual level relational support from teachers and peers that were incorporated into the model. Together, students’ individual level variables reduced the unexplained variance by 14% at level 1, and classroom level variables reduced the unexplained variance by 30% at level 2.

#### Anxious Solitary Behavior

At the individual level, gender, social development and social demonstration-avoidance goals, and relational support from teachers and peers were significantly associated with anxious solitary behavior. Girls reported higher levels of anxious solitary behavior than boys (β = 0.21, *p* < 0.05). Social development goals were negatively associated with anxious solitary behavior (β = −0.12, *p* < 0.05), and social demonstration-avoidance goals were positively associated with anxious solitary behavior (β = 0.22, *p* < 0.001). Perceived teacher support and peer support demonstrated the negative associations with anxious solitary behavior (β = −0.10, *p* < 0.05; β = −0.19, p < 0.001). Students’ individual level variables reduced the unexplained variance by 9% from the null model. At the classroom level, classroom level means for teacher support and peer support were unrelated to students’ anxious solitary behavior (β = −0.05, *p* = 0.15; β = −0.10, *p* = 0.19).

## Discussion

Adolescents perceive and engage differently in their social interactions and relationships with others. Although as a group they may have shared knowledge and understanding about their social worlds, the personal meanings of their interpersonal environments varies for individuals. Psychological processes occur in tandem with the visible social interactions present in the social setting, and individual differences in psychological processes are associated with youth’s different social behaviors and adjustment (Crick and Dodge, 1994). The results of the current study show that youth have significant differences in psychological processes of social goals and perceived relatedness, and these differences matter for youth’s social behavior. Specifically, findings emphasized that youth’s social goals and perceived relational support from teachers and peers made additive and interactive contributions to their emerging social behavior. The results provide important evidence as to which aspects of youth’s cognitive representations and perceived relational features may operate as protective or risk factors separately or in conjunction with each other.

Consistent with previous findings (Ryan et al., 2012; Shin and Ryan, 2012), social achievement goals set in motion different processes for how youth approached, withdrawed, and functioned in social situations. Social development goals were positively related to prosocial behavior and negatively related to relational aggression and anxious solitary behavior. Social demonstration-approach goals were positively related to overt and relational aggression, and social demonstration-avoidance goals were positively related to anxious solitary behavior. These results suggest that a focus on the growth of social relationships and social competence leads to a positive orientation toward youth’s social worlds that sets in motion adaptive beliefs and favorable behaviors. In contrast, a focus on the appearance of the self, achieving social status and recognition or avoiding negative social judgments could be easily associated with maladaptive behavior (Ryan et al., 2012).

Current findings with Asian early adolescents indicate that, in general, overall patterns of the associations between social goals and social behavior are similar between youth in Asia and the Western populations. Thus, the relations between social goals and social behavior seem to be universal during this phase of development. However, results showed that Asian youth adopted higher social development goals (*M* = 4.12) compared to youth in the West (*M* = 3.78; see Ryan and Shim, 2008), whereas social demonstration goals were similarly endorsed by youth in both contexts. In addition, social demonstration-avoidance goals were more strongly associated with anxious solitary behavior among Asian youth (*r* = 0.18) compared with youth in the West (*r*s = 0.09–0.16; see Ryan and Shim, 2008; Shin and Ryan, 2012). These findings indicate that collectivist orientations that emphasize harmonious relationships and interdependence may have implications for what type of social goals youth pursue and the consequences of endorsing particular social goals. Social goals focused on the growth of friendships may be more dominant and social goals focused on avoiding negative evaluations may be more maladaptive in collectivist cultures due to a focus on others more than the self (Markus and Kitayama, 1991; Makara, 2019). However, since there is not yet sufficient evidence to draw strong conclusions, these differences should be interpreted with some caution.

Beyond social achievement goals, youth’s perceived relational support from teachers and peers were negatively associated with subsequent overt and relational aggression as well as anxious solitary behavior. Further, perceived teacher support was additionally positively associated with prosocial behavior. These results conform to the view that personal relational experiences function as relational supports or stressors (Ladd and Burgess, 2001) and suggest that social relatedness affects emerging social adjustment beyond the impact of cognitive factors, such as social achievement goals. Evidence was found for perceived relational support from both teachers and peers. Results for testing teacher and peer support simultaneously indicated that relational support from both operated as relational supports, while teacher support made a unique contribution to youth’s prosocial behavior over and above the substantial contribution of peer support.

At the same time, results indicated that the effects of social goals on social behavior were moderated by the levels of perceived teacher support. Findings were congruent with the view that relational support compensates for dysfunctions that are linked with the risk factors (Hughes et al., 1999). Results suggested that relational protective factors such as teacher support was negatively linked to the levels of aggression primarily among youth who had high social demonstration-approach goals, and these moderated linkages were found for both overt and relational aggression. Current results add to the growing evidence that peers are substantial for youth’s social adjustment, while also emphasizing the continued significance of teacher support (Shin and Ryan, 2017). The fact that perceived teacher support made a unique contribution to youth’s prosocial behavior and attenuated the magnitude of the associations between social demonstration-approach goals and their subsequent aggression suggests that teacher support matters for early adolescents. The lack of evidence for moderating effects of peer support suggests that having relational support from peers is not enough to develop positive social adjustment when their perceptions of teacher support are low. Therefore, effective interventions may need to aim for developing positive relationships with both teachers and peers, and more individualized interventions should be taken place for these youth, even when they are surrounded by multiple friends.

It should be noted that although we did not find a significant association between peer support and prosocial behavior and the moderating effects of peer support at the individual level, results for the classroom-level indicated that early adolescents’ collective perceptions of peer support were positively associated with prosocial behavior. Therefore, the classroom level peer support made a unique contribution to youth’s prosocial behavior over and above the substantial contribution of youth’s individual level relational support from teachers and peers. This suggests that youth’s perceived relational climate (e.g., how youth collectively perceive and characterize their overall class climate) contributes to their levels of prosocial behavior. It is also possible that our measure of peer support could only capture youth’s perceptions of their immediate peer interactions or class climate rather than their dyadic relationship with peers. Compared to teacher support measure that ask youth to report on their individual relationship with their teacher (e.g., “My teacher tries to help me when I am sad or upset”), our measure of peer support ask youth to report on their perceived interactions with all other peers in class (e.g., “Most students are friendly to each other). Given the limited body of research that use both individual and class-level indicators of perceived relational support, further research that incorporate individual and class characteristics to clarify their relative associations with youth’s social behaviors is needed to confirm these associations.

As anticipated, the levels of aggression and anxious solitary behavior and the degree to which perceived teacher support played a role in aggression was affected by gender. Boys reported higher overt aggression than girls, and girls reported higher anxious solitary behavior than boys. Also, moderation analyses showed that perceived teacher support was negatively related to the levels of overt aggression, especially for boys. At large, girls report higher levels of social relatedness than boys in general, and boys are less likely than girls to have close and positive relationships with their teachers (Jerome et al., 2009). Thus, potentially unique experiences of having close relationships with their teacher may serve as stronger protective factors and have a more salient impact on their social adjustment for boys compared with girls. Collectively, the current results emphasize that given that boys generally perceive less relational support from their teacher than do girls (Gest et al., 2005), having social relatedness from a teacher will be especially beneficial for boys.

Although the associations between social goals, perceived relational support, and social behavior were affected by gender, we did not find evidence that these associations were varied by grade. This could be because our cohorts were only 1 year apart. Perhaps grade level differences become stronger in middle school. Since we investigated only change over a semester and compared these within-semester processes for early adolescents of fifth and sixth graders in elementary school, we may not detect meaningful developmental characteristics. Future research that follows the same cohort of early adolescents across multiple years would be informative about how adolescents’ broad social-behavioral orientations as moving toward, away from, or against the world change from early adolescence through late adolescence against the backdrop of changing social contexts. Starting with even middle childhood in earlier grades and tracking youth beyond the middle school would provide greater contrasts and expanded insight into the influence of perceived relational support in developmental trajectories of social adjustment.

Although findings of the current study provide many insights, limitations should be noted and addressed in future research. First, all constructs used in the present work stem from self-reported measures. With the focus on youth’s cognitive representations and perceptions, using self-reported measures could provide important insights into psychological processes of social goals and perceived relational support, that appear to contribute to varied social behaviors. However, relying on only self-reported measures could have inflated the associations between the constructs. Obtaining reports of youth’s behavior from additional sources such as teachers or peers could enhance the measurement validity of the construct and provide a different perspective. Second, to consider the full social context that youth experience in the class, the current study focused on perceived relational support from teachers and peers. However, to better understand youth’s divergent developmental paths, examining proximal relationships with adolescents and their other significant social partners, such as parents at home, is needed. Given multiple social contexts work together to shape individual differences in adjustment, future research could examine the joint implications of parent, teachers, and peers in youth’s social adjustment.

## Conclusion

Adolescents’ social goals have received much attention due to their influence on youth’s adjustment. Although the linkages between social goals and behavior have been extensively examined, scant attention has been paid to the role of social relatedness as a possible moderator. The current study examined if youth’s social goals and perceived relational support had the additive and interactive effects on their social behavior. Findings make several contributions to the literature. Results indicate that social goals and perceived relational support make additive contributions to youth’s social behavior. Social development goals and perceived relational support were positively related to prosocial behavior and were negatively related to aggression and anxious solitary behavior, whereas social demonstration-approach goals were positively related to aggression. Results also indicate the evidence of interactive effects. Perceived teacher support was negatively related to the levels of aggression primarily among youth who have high social demonstration-approach goals. Overall, findings indicate that individual differences in psychological processes of social goals and perceived relatedness matter for youth’s social adjustment, and they emphasize the need to consider adolescents’ social goals in conjunction with their perceptions of the relational features of their interpersonal environments.

## Data Availability Statement

The datasets used in this study are not readily available because all data are treated with complete confidentiality. Descriptive information can be provided upon request.

## Ethics Statement

The studies involving human participants were reviewed and approved by the Oklahoma State University, United States. Informed consents and assents were obtained from all participants included in the study. Students signed an assent form indicating that they understood the conditions and wanted to participate prior to starting the survey. The students’ parents received a letter explaining what was involved in participating in the research, and if they did not want their children to participate, they could opt out by contacting the school; otherwise, students took part in the study.

## Author Contributions

HS conceived of the study, did the analyses and interpreted the data, and drafted the manuscript.

## Conflict of Interest

The author declares that the research was conducted in the absence of any commercial or financial relationships that could be construed as a potential conflict of interest.

## Publisher’s Note

All claims expressed in this article are solely those of the authors and do not necessarily represent those of their affiliated organizations, or those of the publisher, the editors and the reviewers. Any product that may be evaluated in this article, or claim that may be made by its manufacturer, is not guaranteed or endorsed by the publisher.
