# Supplementary material for: Early Adolescents’ Social Achievement Goals and Perceived Relational Support: Their Additive and Interactive Effects on Social Behavior
Source: Front Psychol. 2021 Dec 6;12:767599. doi: 10.3389/fpsyg.2021.767599 (PMC8687115; doi:10.3389/fpsyg.2021.767599)
Supplement: Supplementary file 1 [file Data_Sheet_1.docx]

**­­Appendix A**

**Level 1**

Social Behavior_wave 2_ = β_0j_ + β_1j_(Gender_ij_) + β_2j_(Grade_ij_) + β_3j_(Social Development_ij_) + β_4j_(Social Demonstration-Approach_ij_) + β_5j_(Social Demonstration-Avoidance_ij_) + β_6j_(Teacher Support_ij_) + β_7j_(Peer Support_ij_) + r_ij_

**Level 2**

β_0j_ = γ_00_ + γ_01_(Teacher Support Mean_ij_) + γ_02_(Peer Support Mean_ij_) + u_0j_

β_1j_ = γ_10_ + γ_11_(Teacher Support Mean_ij_) + γ_12_(Peer Support Mean_ij_) + u_1j_

     β_2j_ = γ_20_ + γ_21_(Teacher Support Mean_ij_) + γ_22_(Peer Support Mean_ij_) + u_2j_
    β_3j_ = γ_30_ + γ_31_(Teacher Support Mean_ij_) + γ_32_(Peer Support Mean_ij_) + u_3j_
    β_4j_ = γ_40_ + γ_41_(Teacher Support Mean_ij_) + γ_42_(Peer Support Mean_ij_) + u_4j_

     β_5j_ = γ_50_ + γ_51_(Teacher Support Mean_ij_) + γ_52_(Peer Support Mean_ij_) + u_5j_

     β_6j_ = γ_60_

     β_7j_ = γ_70_
